# Characterizing the breast cancer lipidome and its interaction with the tissue microbiota

**DOI:** 10.1038/s42003-021-02710-0

**Published:** 2021-10-27

**Authors:** Natasa Giallourou, Camilla Urbaniak, Scarlett Puebla-Barragan, Panagiotis A. Vorkas, Jonathan R. Swann, Gregor Reid

**Affiliations:** 1 Department of Metabolism, Digestion, and Reproduction, Faculty of Medicine, Imperial College London, London, UK; 2 Center of Excellence in Biobanking and Biomedical Research and Molecular Medicine Center, University of Cyprus, Nicosia, Cyprus; 3 NASA Jet Propulsion Laboratory, California Institute of Technology, Pasadena, CA USA; 4 ZIN Technologies Inc., Middleburg Heights, OH USA; 5 Department of Microbiology and Immunology, Western University, London, ON Canada; 6 Lawson Health Research Institute, 268 Grosvenor St., London, ON N6A 4V2 Canada; 7 Institute of Applied Biosciences, Centre for Research and Technology Hellas, 570 01 Thessaloniki, Greece; 8 School of Human Development and Health, Faculty of Medicine, University of Southampton, Southampton, UK

**Keywords:** Systems biology, Medical research

## Abstract

Breast cancer is the most diagnosed cancer amongst women worldwide. We have previously shown that there is a breast microbiota which differs between women who have breast cancer and those who are disease-free. To better understand the local biochemical perturbations occurring with disease and the potential contribution of the breast microbiome, lipid profiling was performed on non-tumor breast tissue collected from 19 healthy women and 42 with breast cancer. Here we identified unique lipid signatures between the two groups with greater amounts of lysophosphatidylcholines and oxidized cholesteryl esters in the tissue from women with breast cancer and lower amounts of ceramides, diacylglycerols, phosphatidylcholines, and phosphatidylethanolamines. By integrating these lipid signatures with the breast bacterial profiles, we observed that *Gammaproteobacteria* and those from the class *Bacillus*, were negatively correlated with ceramides, lipids with antiproliferative properties. In the healthy tissues, diacylglyerols were positively associated with *Acinetobacter, Lactococcus, Corynebacterium, Prevotella* and *Streptococcus*. These bacterial groups were found to possess the genetic potential to synthesize these lipids. The cause-effect relationships of these observations and their contribution to disease patho-mechanisms warrants further investigation for a disease afflicting millions of women around the world.

## Abstract (web summary)

Giallourou, Urbaniak et al. investigated the lipidome and microbiome of breast tissues adjacent to breast tumors and control, healthy breast tissue, by examining both bacterial colonization and lipid metabolism. As a result, the authors propose a relationship between breast oncobiosis and the breast lipidome.

## Introduction

Breast cancer is a multifaceted disease characterized by a complex interplay between genomic and physiological interactions at the tumor site, which can result in different prognostic attributes and therapeutic implications. It is the most commonly diagnosed cancer amongst women worldwide affecting up to one in eight during their lifetime, but its etiology is still not completely understood and causal pathways have been difficult to delineate. Recent technological and scientific advances in the field of ‘omics’ have uncovered previously unknown features of disease ontology further advancing our knowledge on the role of genetics and the environment in breast cancer^1^.

Accumulating evidence suggests that bacterial communities within breast tissue could be an additional environmental factor contributing to cancer development^2–12^. We have previously shown that human breast tissue is not sterile and contains diverse bacterial communities whose composition differs in normal tissue between healthy women and those with breast cancer^2,3^. However, it is unclear if differences in the breast microbiome are a cause or consequence of the pathology.

The biomolecular processes occurring within tumors differ from those occurring in normal tissue and a variety of cancers share common metabolic features. Hence, dysregulated metabolism constitutes one of the established hallmarks of cancer^13^. Reprogrammed tumor cell metabolism aims to modify cellular fitness in a manner that presents a selective advantage during malignancy. Biosynthesis and turnover of lipids are considerably increased in tumor cells to meet the anabolic requirements and redox needs of the proliferating tissue^14^. Lipids are also shuttled towards the formation of cellular membranes and signaling molecules. Shifts in cellular lipid profiles thereby influence breast cancer metabolism and progression^14^. Bacteria also process a variety of lipid species as an energy source and obtain the building blocks required for the synthesis of their cell envelope. The intestinal microbiota has been shown to modulate host lipid metabolism in the gut and at the systemic level through direct and indirect interactions^15^. Variation in the lipid content of a host environment can therefore influence the microorganisms present and conversely, the activity of the different microbial inhabitants can modify the lipid landscape of that niche and its visibility and bioactivity for the host.

The aim of this study was to characterize the lipidomic signatures of breast tissue collected adjacent to breast tumors and compare it to those collected from healthy individuals who were disease-free. In addition, the relationship between the breast microbiota and the surrounding lipid profiles was explored as well as the effect of breast cancer on these associations.

## Results

### Characterizing the lipidomic profile of breast tissue from healthy women and breast cancer patients

Fresh breast tissue was collected from 61 women undergoing breast surgery at St. Joseph’s Health Centre in London, Ontario, Canada. Forty-two women underwent lumpectomies or mastectomies for breast tumors, while 19 were free of disease (“healthy”) and underwent either breast reductions or enhancements. For those women with tumors, the tissue obtained for analysis was collected outside the marginal zone, approximately 5 cm away from the tumor. This is termed ‘tumor-adjacent-normal’ tissue. Tissue collected from healthy women is referred to as ‘healthy-normal’ tissue. None of the subjects had been on antibiotics for at least 3 months prior to collection. The lipidomic profiles of the tissue extracts were measured by ultra-performance liquid chromatography-mass spectrometry (UPLC-MS) in both positive and negative electrospray ionization (ESI) modes.

**Fig. 1:**
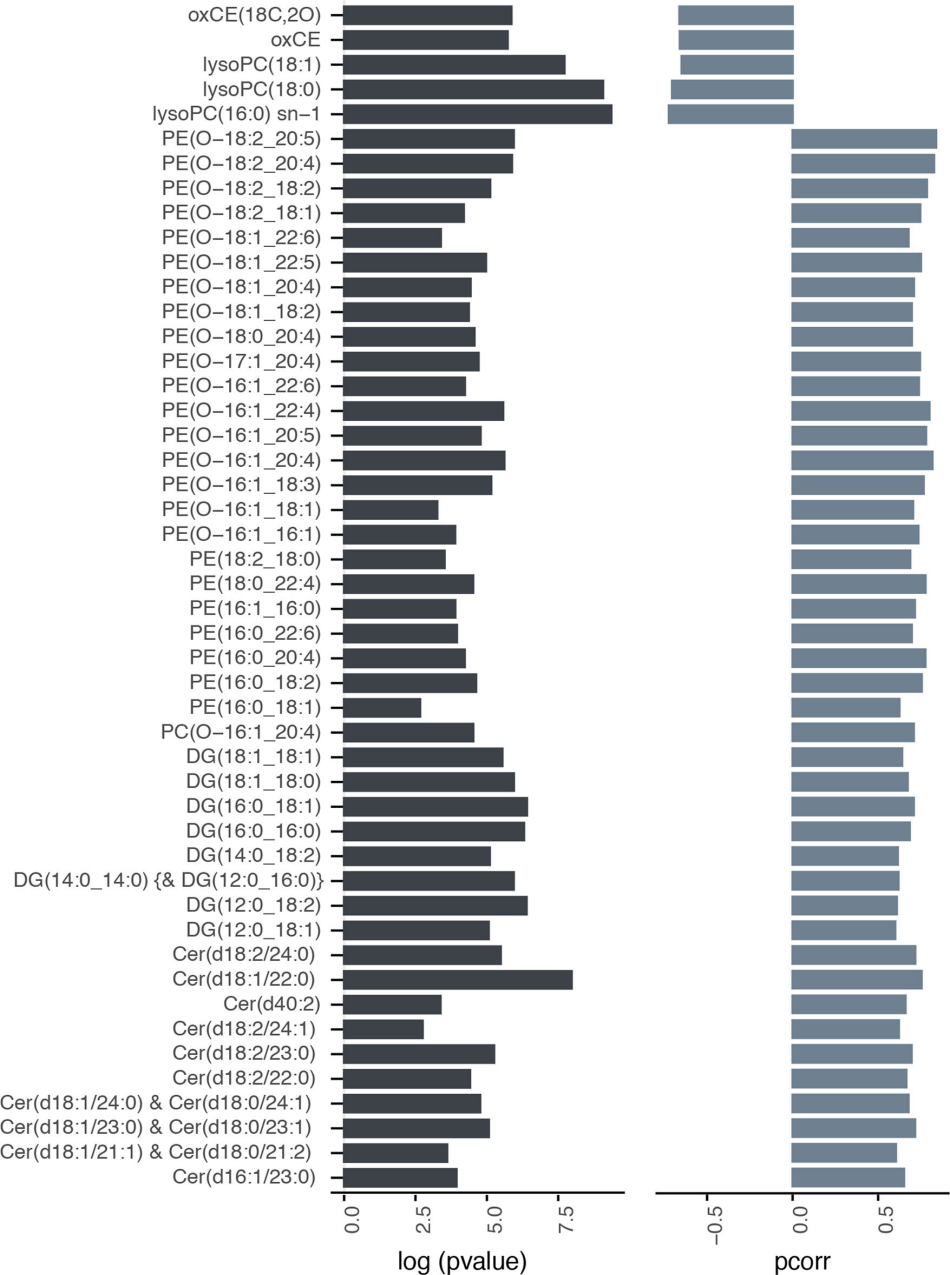
Discriminant lipid species between healthy-normal and tumor-adjacent-normal tissues. Lipid species found in the OPLS-DA models (ESI+ and ESI−) to be significantly different in breast tissue collected from healthy women (*n* = 19) and those with breast cancer (*n* = 42). Left panel, bar plot presenting the log(*p* value) from the *t* test (two-tailed; assuming unequal variance) comparing the two groups. Right panel, bar plot indicating the *p*(corr) values obtained from the OPLS-DA models built on the lipidomic profile (negative *p*(corr) indicates the lipid feature is more abundant in the tissue from cancer patients compared to healthy controls). Top five features in the list are uniquely present in tissues from cancer patients. Cer ceramide, DG diacylglycerol, lysoPC lysophosphatidylcholine, oxCE oxidized cholesteryl ester, PC phosphatidylcholines, PE phosphatidylethanolamines.

Orthogonal projection to latent structures-discriminant analysis (OPLS-DA) models were constructed to assess differences in the lipid profiles of healthy-normal tissue and tumor-adjacent-normal tissue. Lipid features were Pareto-scaled, and log-transformed and model validation was carried out using cross-validation-analysis of variance (CV-ANOVA) testing. From these models, clear differences were observed in the breast lipids between the two study groups measured by both ESI modes. These models displayed high predictive scores, indicating distinctive lipid phenotypes in healthy-normal tissue and tumor-adjacent-normal tissue (ESI+ mode model diagnostics: *R*^2^X = 0.361, *R*^2^Y = 0.784, *Q*^2^Y = 0.505, *P* value = 2.66e-09; ESI− mode model diagnostics: *R*^2^X = 0.303, *R*^2^Y = 0.659, *Q*^2^Y = 0.392, *P* value = 1.94e-07). For putative biomarker extraction, appropriate feature selection, and lipid structure annotation of candidate biomarkers, the workflow described by Vorkas et al. was followed^16,17^. A total of 48 unique lipid species were annotated, which were found to be statistically significantly different between the two tissue groups (Fig. 1). The lipid profile of tumor-adjacent-normal tissue was characterized by the accumulation of lysophosphatidylcholines (LysoPCs) and oxidized cholesteryl esters (oxCEs). Such features were absent from the healthy-normal tissues. Conversely, breast tissue from healthy women presented a more diverse lipid profile with higher intensities of several lipid classes such as diacylglycerols (DG), ceramides (Cer), phosphatidylcholines (PC), and phosphatidylethanolamines (PE).

### Variation in the breast microbiome between healthy controls and breast cancer patients

Comprehensive analysis of the microbiome of the same breast tissues has previously been performed using 16S rRNA gene sequencing analysis and the results are described in Urbaniak et al.^2,3^. For the purposes of this manuscript, a subset of these bacterial profiles was investigated where matching lipidomics data existed (16 healthy controls, 32 breast cancer patients). Using ALDEx2, bacterial taxa were identified whose relative abundances significantly differed between the two study groups. Tumor-adjacent-normal tissue was found to contain higher relative abundances of *Staphylococcus, Bacillus*, and *Gammaproteobacteria* (unclassified), while healthy-normal tissue harbored higher abundances of *Corynebacterium, Acinetobacter, Prevotella, Gammaproteobacteria* (unclassified), and *Lactococcus* (Table 1).

**Table 1:** Bacterial relative abundances in normal and tumor-adjacent-normal tissue.

Genus	Median log2 RA Cancer	Median log2 RA Healthy	Effect size	*P* value	*P*_adj_
*Corynebacterium*	−3.05078	0.55456	0.93460	0.00002	**0.00024**
*Acinetobacter*	3.52399	4.99866	0.89468	0.00004	**0.00047**
*Prevotella*	−0.07806	2.23876	0.83597	0.00010	**0.00085**
*Gammaproteobacteria* (unclassified)	−3.41404	0.24559	0.83370	0.00022	**0.00147**
*Prevotella*	−2.19046	0.61126	0.83049	0.00048	**0.00297**
*Lactococcus*	1.80420	3.84987	0.74354	0.00003	**0.00038**
*Lactococcus*	1.21785	3.44230	0.72191	0.00041	**0.00267**
*Prevotella*	−3.88765	−1.18212	0.67504	0.00204	**0.00919**
*Lactococcus*	−0.23240	3.06471	0.56543	0.00320	**0.01553**
*Prevotella*	−3.43107	−1.36356	0.49660	0.03067	0.08866
*Streptococcus*	−0.77006	1.46508	0.42017	0.04131	0.12656
*Micrococcus*	0.52029	1.11744	0.36611	0.03150	0.09472
*Staphylococcus*	−1.35312	−3.22940	−0.44771	0.03066	0.08570
*Staphylococcus*	2.57149	1.37032	−0.45353	0.03536	0.11197
*Lactobacillus*	2.73155	1.64036	−0.47398	0.02433	0.08437
*Propionibacterium*	4.31073	3.43269	−0.48797	0.03016	0.10008
*Bacillus*	−2.22084	−4.77999	−0.51615	0.02394	0.06333
*Bacillales* (unclassified)	−1.45938	−4.20697	−0.51839	0.01784	0.05692
*Bacillus*	−2.39633	−5.17174	−0.55899	0.01690	0.05183
*Staphylococcus*	1.32550	−1.53964	−0.66019	0.00057	**0.00348**
*Bacillus*	1.14347	−2.40476	−0.79326	0.00020	**0.00140**
*Gammaproteobacteria* (unclassified)	0.05306	−3.91697	−0.98396	0.00002	**0.00020**
*Bacillus*	4.56707	0.08631	−1.32125	0.00000	**0.00000**

Median log2 relative abundances (RA) and effect sizes of different genera in healthy-normal tissue and tumor-adjacent-normal tissue. Negative effect size value indicates a higher relative abundance of genera in tumor-adjacent-normal tissue compared to healthy-normal tissue.

Bolded values indicate bacterial genera found to significantly differ between the study groups after adjusting for multiple testing.

### Elucidating microbial−lipid interactions in breast tissue

**Fig. 2:**
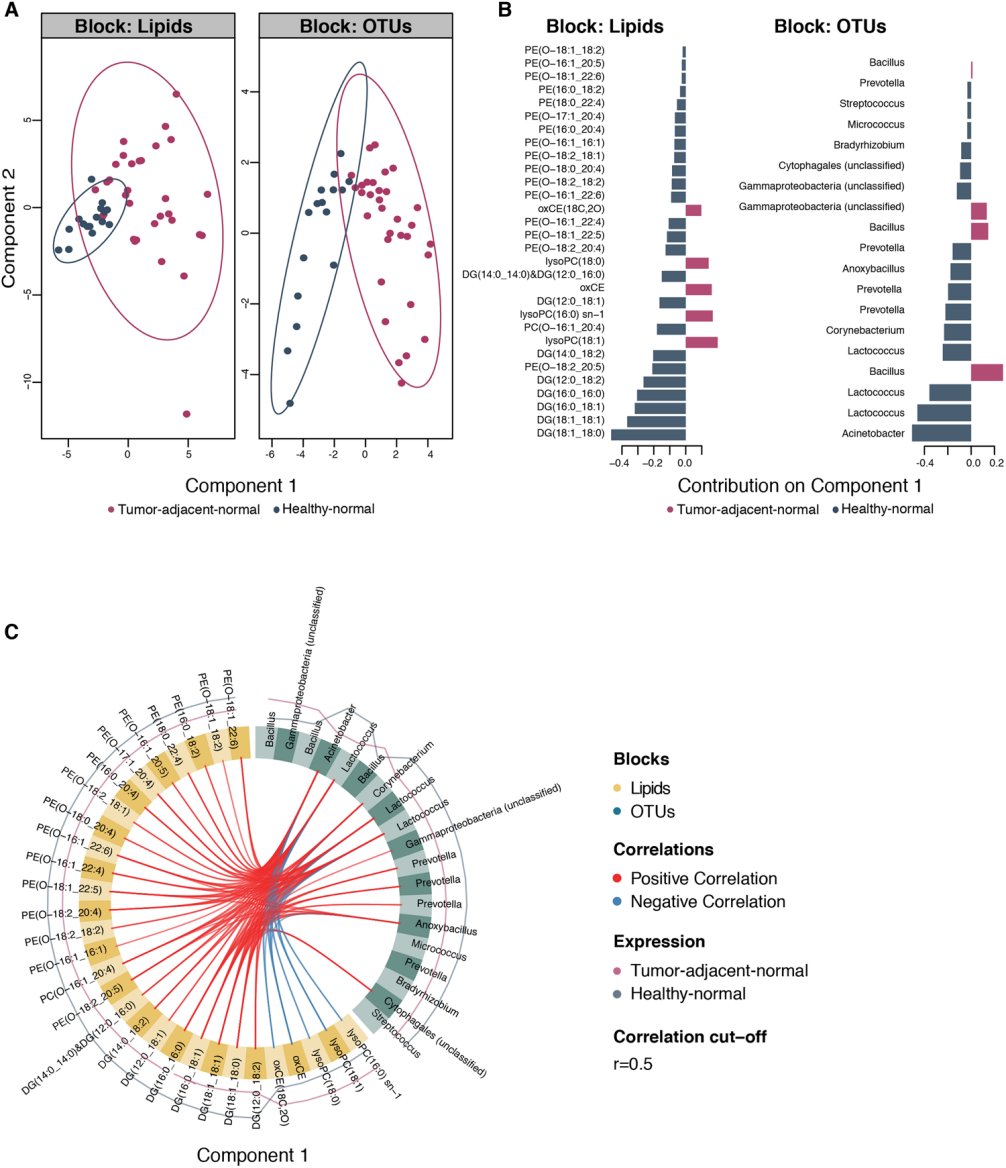
Integrative analysis of lipid and bacterial profiles in healthy-normal and tumor-adjacent-normal tissues. Integration of lipid and bacterial profiles using multi-block sparse-partial least-squares-discriminant analysis (sPLS-DA). **A** Score plots showing samples from each data set projected in latent space. Tumor-adjacent-normal (*n* = 32) and healthy-normal tissues (*n* = 16) are discriminated along component 1. **B** Loading weights of each of the selected discriminant variables on component 1 for each data block. **C** Circos plot showing correlations between variables in the different data sets along component 1, derived from the DIABLO model using sPLS-DA. DG diacylglycerol, lysoPC lysophosphatidylcholine, oxCE oxidized cholesteryl ester, PE phosphatidylethanolamines.

Relationships between lipids and bacterial taxa were further investigated using a multi-block sparse-PLS-DA (sPLS-DA) approach to identify the most discriminatory bacterial OTUs and lipids between healthy-normal (*n* = 16) and tumor-adjacent-normal tissues (*n* = 32). Tumor-adjacent-normal tissues were discriminated from healthy controls on the first component of the model for both lipid and OTU data sets (Fig. 2A). The importance of each variable in the process of tissue classification is shown in Fig. 2B. Lysophosphatidylcholines and oxidized cholesteryl esters from the lipid block and *Bacillus* and *Gammaproteobacteria* (unclassified) from the bacterial block were the most important variables for discriminating tumor-adjacent-normal tissue from the healthy-normal tissue. The circos plot derived from the sPLS-DA model displays the features selected from the model to best classify the phenotypes (Fig. 2C). The links between the two data sets indicate a strong inverse correlation between *Acinetobacter* and *Lactococcus*, which were more abundant in tissues from healthy individuals, and lysophosphatidylcholines and oxidized cholesteryl esters. A strong positive correlation was observed between *Acinetobacter, Lactococcus, Corynebacterium, Prevotella, Anoxybacillus,* and *Cytophagales* (unclassified) and phosphatidylethanolamines, diacylglycerols and ceramides, all of which were more abundant in the healthy-normal tissues.

**Fig. 3:**
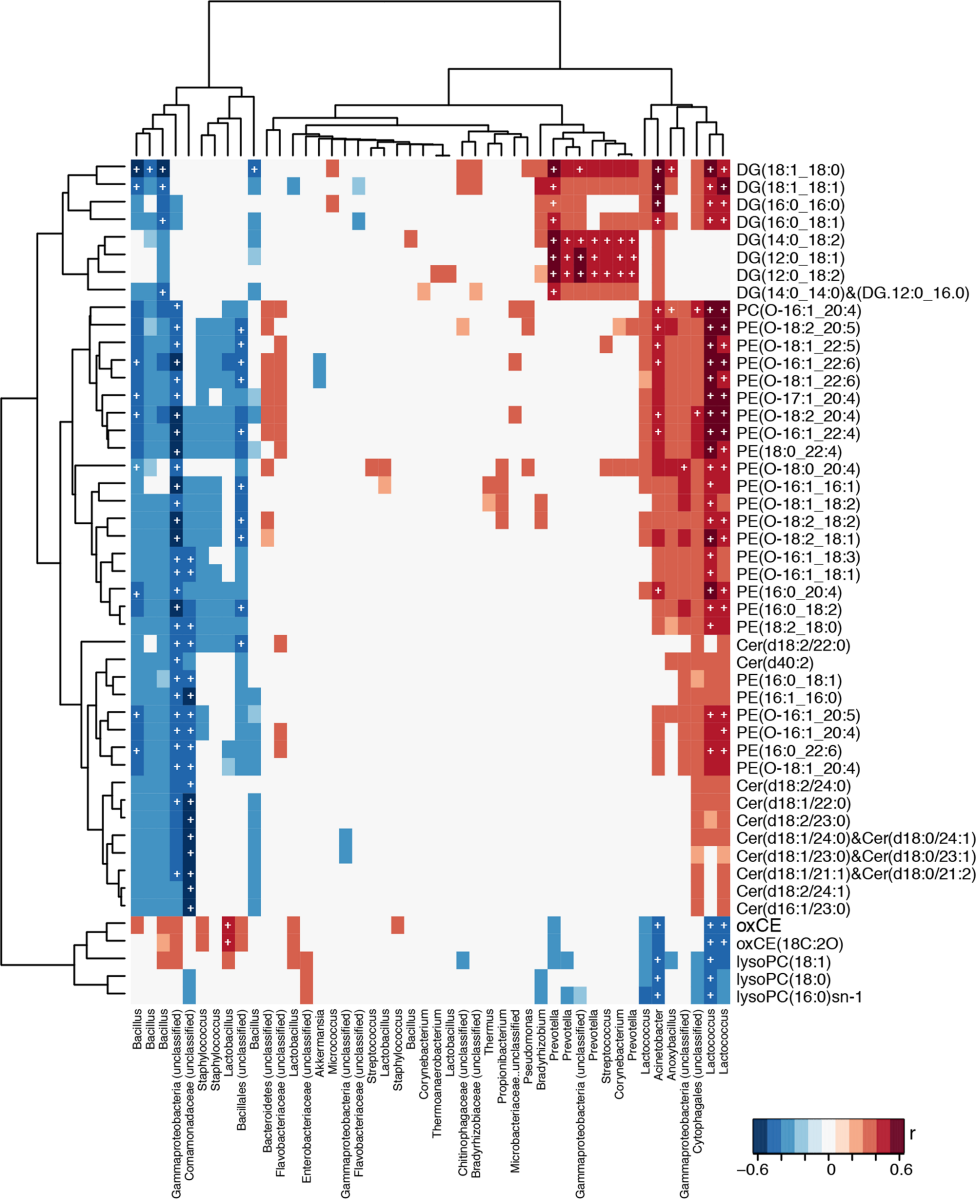
Correlations between discriminant lipid and bacterial OTUs. Pearson’s correlation analysis identifying associations between lipids and bacterial OTUs in healthy-normal (*n* = 16) and tumor-adjacent-normal tissues (*n* = 32). Significant associations after FDR correction (*p* < 0.05) are denoted with a white cross. Cer ceramide, DG diacylglycerol, lysoPC lysophosphatidylcholine, oxCE oxidized cholesteryl ester, PE phosphatidylethanolamines.

Integrated analysis of the microbial and lipidomic data was also performed without prior variable selection using Pearson’s correlation coefficients (Fig. 3). Results from this analysis were evaluated with and without false discovery rate (FDR) correction. *Gammaproteobacteria* (unclassified) and *Comamonodaceae* were significantly inversely correlated with the abundance of Cer and PEs. *Staphylococcus* did not associate with ceramides but exhibited negative correlations with PEs. *Bacillus* was negatively associated with the majority of lipid features that were found in higher amounts in the healthy-normal tissues, but few associations survived FDR correction. *Enterobacteriaceae* were positively correlated to LysoPCs and *Bacteroidetes* (unclassified) were positively associated with several PEs but none of these associations remained significant following FDR correction. *Lactococcus* and *Acinetobacter* were inversely associated with LysoPCs and oxCEs, lipids found to be higher in tumor-adjacent normal tissues. The same OTUs were positively associated with PEs and some DGs. *Prevotella*, *Streptococcus* and *Corynebacterium* were positively correlated only to DGs.

### Genetic potential of discriminatory breast tissue microbiota for lipid biosynthesis and metabolism

**Fig. 4:**
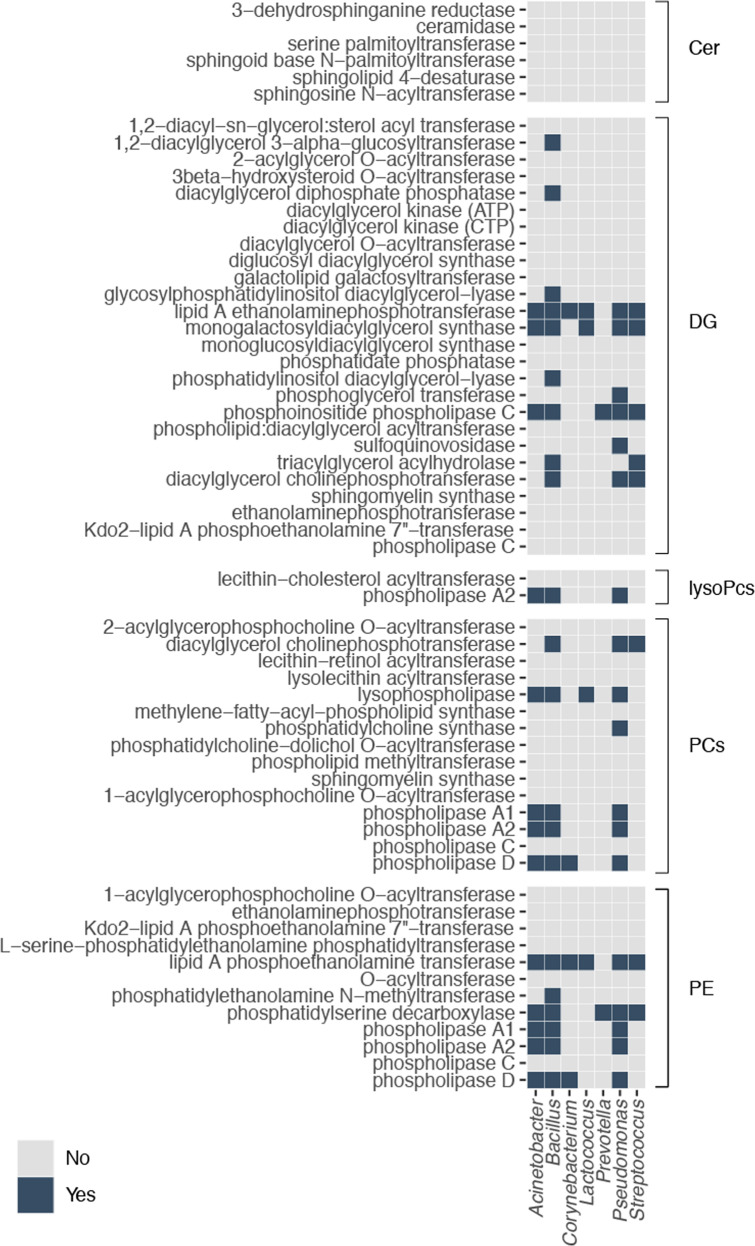
Enzymatic potential of bacterial genera for lipid biosynthesis. Heatmap describing the presence or absence of the genes required for the biosynthesis of lipids in the bacterial genera identified to differ between the study groups. Enzymes are divided according to the biosynthetic pathway. Blue signifies that the gene required for the synthesis of the enzyme of interest has been annotated in the genome of the genus presented on the *X* axis. Cer ceramide, DG diacylglycerol; lysoPC, lysophosphatidylcholine; oxCE, oxidized cholesteryl ester; PC, phosphatidylcholines; PE, phosphatidylethanolamines.

The Kyoto Encyclopedia of Genes and Genomes (KEGG)^18–20^ was used to identify the enzymes responsible for the biosynthesis of Cer (pathway module: M00094), DGs (entries: C00641 and C00165), PCs (entry: C00157), and PEs (entry: C00350). The enzymes required for the biosynthesis of LysoPCs were identified based on a literature search^21^. In total, 5 enzymes were identified that can synthesize Cer, 26 for DGs, 2 for lysoPCs, 15 for PC, and 12 for PE (Fig. 4).

Using the EnzymeDetector^22^ tool of the enzyme repository BRENDA^23^ (www.brenda-enzymes.org), the presence of genes for these enzymes of interest in the genomes of strains of *Acinetobacter, Bacillus, Corynebacterium, Lactococcus, Prevotella, Pseudomonas*, and *Streptococcus* was explored. Ceramidase, which is responsible for the degradation of Cer, was also included in the analysis. The presence of any of these indicates the genomic potential of each genera to synthesize the specific lipid group indicated. A heatmap is provided in Fig. 4 with categorical values denoting the presence or absence of the genes responsible for the enzymes of interest. In the case of the biosynthesis of DGs, the biosynthetic potential was identified in *Acinetobacter*, *Bacillus*, *Corynebacterium*, *Lactococcus*, *Prevotella*, *Pseudomonas*, and in *Streptococcus*. For lysoPCs, one enzyme was identified for *Acinetobacter*, *Bacillus*, *Corynebacterium*, and *Pseudomonas*. Except for *Prevotella*, all of the genera analyzed had at least one enzyme required for the synthesis of PC. All of the genera have the genetic potential to synthesize PE. None of the analyzed genera had the genes required for the biosynthesis of Cer. The gene required for the expression of ceramidase was not identified in any of the genomes of the genera analyzed.

## Discussion

This research has identified distinct lipid signatures in tumor-adjacent-normal breast tissue collected from breast cancer patients versus healthy controls. This provides further evidence that altered cellular lipid metabolism occurs in close proximity to a tumor, an area where microbes are known to inhabit. Tumor-adjacent-normal tissue contained higher amounts of oxCEs and LysoPCs and lower amounts of various DGs, PEs, and Cer compared to healthy-normal tissue. Significant associations were also observed between different bacterial groups measured in the breast tissue and the lipids present. These relationships were altered by the presence of a tumor and their potential to contribute to breast cancer pathomechanisms and influence cancer progression warrant further investigation.

Several long fatty amide-chain Cer (fatty amide chains ranging from 21 to 24 carbons) were less abundant in tumor-adjacent-normal tissue compared to tissue sampled from healthy individuals. Ceramides are antiproliferative^24^ molecules that have been shown to mediate cell death in cancer by inducing apoptosis^25^. Alternatively, Cer can be metabolized to sphingosine-1-phosphate (S1P), which has been attributed with a critical role in breast cancer^26^ as it is associated with the promotion of cell proliferation and survival. The findings presented here are based on tissue adjacent to the tumor (approximately 5 cm away) and the lower abundance of ceramides may reflect increased migration of these molecules into the tumor tissue. This may facilitate cancer progression by impairing the control of cell proliferation and the mechanisms that arrest growth^27^. Consistent with this, previous studies have shown breast tumors contain greater amounts of Cer and S1P compared to healthy tissue from the same individuals^28^. Tumors have also been found to have higher expression of genes involved in all three ceramide biosynthesis pathways^29^.

We previously reported that the breast microbiome of these tissues varied between healthy-normal tissue and that collected adjacent to a tumor. Interestingly, the microbiota was comparable between the tumor and adjacent tissue from the same individual. Tissue from breast cancer patients contained a greater abundance of *Bacillus*, *Staphylococcus*, *Enterobacteriaceae* (unclassified), *Comamondaceae* (unclassified), and *Bacterioidetes* (unclassified) and a lower abundance of *Prevotella*, *Lactococcus*, *Streptococcus*, *Corynebacterium*, and *Micrococcus* compared to the healthy individuals. Similar findings have been reported by others, noting differences in the breast microbiome between healthy individuals and those with breast cancer and no, or minimal, differences between paired normal and tumor tissue from the same individual (reviewed in ref. ^30^). It is unclear if differences in the lipid profiles between tumor-adjacent-normal and healthy-normal tissues are a result of the bacterial variation in the tissues or rather that lipid differences due to the presence of a tumor drive the bacterial variation. We believe the former is a more feasible explanation, as bacteria are known to produce bioactive molecules that have a profound influence on the host^31–33^. Some of these bacterial metabolites play a role in breast cancer processes and include lithocholic acid^34,35^, short-chain fatty acids^36^, cadaverine^37^, or deconjugated estrogens^38,39^.

Our results are consistent with observations made by others where lipid and fatty acid pathways are upregulated, most likely to be used as sources of energy that support tumor cell growth as well as pathogenesis^40–42^. Specifically, for the Cer, the sphingomyelinase-ceramide system has been implicated with a key role in host responses to many pathogens. Sphingomyelinases and Cer are important for the internalization of pathogens, the induction of apoptosis in infected cells and the release of cytokines^43^. It is feasible that modulation of this biochemical capacity during tumorigenesis could alter the host’s ability to control the local bacterial communities. Conversely, several bacterial species are able to synthesize and/or metabolize Cer^43^. In this study, bacteria from the Gram-positive class, *Bacillus* and the Gram-negative class, *Gammaproteobacteria* were negatively correlated with Cer. *Pseudomonas aeruginosa*, a *Gammaproteobacteria* species, can secrete hemolytic phospholipase C, which can synthesize sphingomyelin from Cer, and alkaline ceramidase, an enzyme that can breakdown Cer^44^ to sphingosine. In addition, *Bacteroides* spp. can produce serine palmitoyltransferase, an enzyme that facilitates the production of sphingolipids, including Cer. Sphingolipids derived from bacteria in the gut have been shown to affect host lipid metabolism. As such, there is potential that the tissue microbiota of the cancer patients could modulate Cer availability in the breast through reduced production and enhanced metabolism of these sphingolipids compared to the healthy controls. It is important to note that, although our pathway analysis did not identify genetic potential to metabolize Cer in any of the genera, it is possible that such genes exist but have not yet been annotated. Given the influence of Cer on tumorigenesis, bacterial-related changes in the tissue abundance of these molecules could have implications for tumor growth and represent an attractive target for further study and interventions.

DGs were also depleted in tumor-adjacent-normal tissues compared to healthy-normal tissue. These DGs are key molecules in lipid metabolism. They are fundamental components of cellular membranes and are involved in cell growth and proliferation serving as modulators of signaling proteins in multiple intersecting pathways^45,46^. They act as second messenger signaling lipids and can activate protein kinase C (PKC), a family of serine/threonine kinases that control important biological processes and are involved in cell cycle regulation, apoptosis, cell survival, and tumorigenesis^47^. There is a large body of evidence suggesting that a number of PKC isoenzymes are involved in carcinogenesis, invasion, and metastasis in many types of cancer including breast cancer^48^. It is possible that, like Cer, DGs are recruited from the surrounding tissue to the tumor site for PKC activation and cancer progression, explaining their lower abundance in these tissues. Several of the DGs depleted in the tumor-adjacent-normal tissue were positively correlated with bacteria associated with healthy-normal tissue. This included *Acinetobacter, Lactococcus, Corynebacterium, Prevotella*, and *Streptococcus*. Inspection of the genomes from these bacterial groups indicated that they all possess the enzymatic capacity for the biosynthesis of DGs. Consistently, various bacterial species isolated from human feces have been shown to produce DGs including *Escherichia coli*, *Bifidobacterium infantis*, and *Clostridium bifermentans*^49^. However, increased abundance of exogenous DGs by the intestinal microbiota has been proposed as a tumor-promoting risk factor^49^. As with Cer, it is plausible that DG availability may also influence the bacterial profiles of the tissue. For example, DGs have been identified to have a role in antibacterial autophagy, an important immune response to invading microbes^50^. The DGs target pathogenic bacteria such as *Salmonella* to the autophagy pathway and induce autophagy via PKC activation.

Tumor-associated tissue was found to contain higher amounts of three lysoPC species compared to healthy-normal tissue. LysoPCs are predominantly derived from phospholipase hydrolysis (usually LPA2; also indicated by sn-1 lysoPC detected) or the lecithin-cholesterol acyltransferase (LCAT) mediated transfer of one fatty acid to free cholesterol (producing cholesteryl ester), from the PC. Increased oxCEs in the tumor-adjacent-normal tissue suggest that increased LCAT-catalyzed production of lysoPCs has occurred in these tissues. Additionally, oxCEs can be directly produced from the hydrolysis of PC hydroperoxides by the LCAT enzyme^51^. The strong positive correlations between lysoPCs and oxCEs found here further support this hypothesis. Intriguingly, both lysoPCs and oxCEs were negatively associated with the *Lactococcus* genus. The mechanisms remain to be explored.

Dysregulation of PE metabolism was indicated by the lower abundance of several PE species in tumor-adjacent-normal tissue, predominantly from the ether-PE subclass. De novo PE synthesis occurs by two main pathways: (a) in the endoplasmic reticulum (ER), by incorporating a PEth (CDP-Eth) to DGs, which constitute the final steps of the Kennedy pathway, and (b) in the mitochondria, using the conversion of phosphatidylserine to PE^52^. There is currently no clear evidence as to the effect of these changes on cancer disease.

Conflicting studies show both positive and negative associations with PEs and cancer, suggesting such relationships may be cell-type or cancer dependent^52,53^. Nonetheless, decreased levels of ether-PEs have been reported in breast cancer tumors compared to adjacent normal tissue^54^. Furthermore, the dysregulated PE moieties from our study showed positive correlations to Cer and inverse associations to oxCEs, in concordance with previous reports of associations between ether-PE species and cholesterol and sphingolipid metabolism^52^.

Notable differences were found in the microbial and lipidomic landscapes of breast tissue collected from healthy women compared to those from patients with breast cancer. These connections were supported by the genetic potential of specific bacteria to synthesize certain lipids. The findings admittedly have limitations in terms of sample size and location (i.e. tumor-adjacent-normal tissue rather than tumor tissue), and do not prove cause and effect. Nevertheless, the integrated analysis performed in this study generates new opportunities for better understanding the biological mechanisms underpinning breast cancer and once again illustrates the need to consider the microbiome in the pathogenesis of this common disease.

## Methods

### Tissue collection and processing

Fresh breast tissue was collected from 61 women (Supplementary Table 1) undergoing breast surgery at St. Joseph’s Hospital in London, Ontario, Canada. Ethical approval was obtained from the Western Research Ethics Board and Lawson Health Research Institute, London, Ontario, Canada. Subjects provided written consent for sample collection and subsequent analyses. Forty-two women underwent lumpectomies or mastectomies for breast tumors, while 19 were free of disease (“healthy”) and underwent either breast reductions or enhancements. For those women with tumors, the tissue obtained for analysis was collected outside the marginal zone, approximately 5 cm away from the tumor. None of the subjects had been on antibiotics for at least 3 months prior to collection.

After excision, fresh tissue was immediately placed in a sterile vial on ice and homogenized within 30 min of collection. Tissue samples were homogenized in sterile PBS using a PolyTron 2100 homogenizer at 28,000 rpm. The amount of PBS added was based on the weight of the tissue in order to obtain a final concentration of 0.4 g/ml. The homogenate was then stored at −80 °C until metabolome analysis.

### Tissue metabolite extraction

Breast tissue samples (40 mg) were combined with 300 μl of pre-chilled chloroform:methanol (2:1) and homogenized using a tissue lyzer. The homogenate was combined with 300 μl water, vortexed and spun (1000 × *g* for 15 min at 4 °C) to separate the aqueous and organic phases into two glass vials. 300 μl water and 300 μl of chloroform:methanol (2:1) were added to the pellet and samples were vortexed and spun again. The aqueous and organic layers were transferred into their respective glass vials. The organic phase supernatants were allowed to evaporate at room temperature in an extractor hood overnight and stored at −40 °C until analysis.

### UPLC-MS analysis of organic extracts

Ultra-performance liquid chromatography coupled to mass spectrometry (UPLC-MS) analysis was performed as described in Vorkas et al.^17^. The organic extracts were subjected to a lipid profiling reversed-phase (RP) UPLC-MS analysis. The evaporated extracts of the breast tissue samples were reconstituted in 500 μl of the solvent mixture:water/acetonitrile (CAN)/isopropanol (ISP) (1:1:2) and then centrifuged at 5000 × *g* for 10 min at 4 °C. The extracts was then transferred into Total Recovery vials (Waters Corp, USA). UPLC separation was conducted using an Acquity UPLC System (Waters Corp, USA) using an Acquity UPLC CSH C18 2.1 × 100 mm, 1.7 μm, column (Waters Corp, USA). The column temperature was set to 55 °C and flow rate at 0.4 mL/min. Mobile phase A consisted of ACN/water (60:40) and mobile phase B consisted of ISP/ACN (90:10). Ammonium formate diluted to a concentration of 10 mM and formic acid to 0.1% was added to both mobile phases A and B. The elution gradient was set as follows: 60–57% A (0.0–2.0 min), 57–50% A (2.0–2.1 min; curve 1), 50–46% A (2.1–12.0 min), 46–30% A (12.0–12.1 min; curve 1), 30–1% A (12.1–18 min), 1–60% A (18.0–18.1 min), 60% A (18.1–20.0 min). Injection volumes of 3 and 7 μl were used for positive and negative ionization modes, respectively. The autosampler temperature was set to 4 °C. Mass spectrometry was performed using a Xevo G2 QTof (Waters MS Technologies, UK) with an electrospray ionization (ESI) source. A quality control pooled sample (QC) was injected every ten samples in order to assess instrument stability and feature reproducibility through the run^55^. Data extraction for both analyses was conducted using the XCMS package^56^ (version 1.34.0) in R programming language (version 2.15.2).

### Microbiome data acquisition

Detailed description of the DNA isolation process, V6 16S rRNA sequencing-PCR amplification and sequence processing, and taxonomic assignment is presented in Urbaniak et al.^3^. (i) DNA isolation: In a tube, 1.2 ml of ASL lysis buffer (QIAamp, DNA stool kit; Qiagen) was added to 400 μl of thawed tissue homogenates together with 400 mg of 0.1 mm diameter zirconium glass beads (BioSpec Products). 800 μl of PBS control and 800 μl of the skin swab control were then added to the same tube. Bead beating (Mini beadbeater 1; BioSpec Products) at 4800 rpm for 60 s at room temperature and then 60 s on ice (repeated twice) was performed for mechanical and chemical lysis of all samples. The suspension was then incubated at 95 °C for 5 min. Subsequent procedures were performed using the Qiagen QIAamp DNA stool kit according to the manufacturer’s protocol, with the exception of the last step, in which the column was eluted with 120 μl of elution buffer. DNA was stored at −20 °C until further use. (ii) V6 16S rRNA gene sequencing: PCR amplification: The genomic DNA isolated from the clinical samples was amplified using barcoded primers that amplified the V6 hypervariable region of the 16S rRNA gene (70 bp long): V6-forward, 5′ACACTCTTTCCCTACACGACGCTCTTCCGATCTnnnn(8)CWACGCGARGAACCTTACC3′; and V6-reverse, 5′CGGTCTCGGCATTCCTGCTGAACCGCTCTTCCGATCTnnnn(8)ACRACACGAGCTGACGAC3′. In the primers, nnnn indicates four randomly incorporated nucleotides, and 8 represents a specific sample barcode sequence. The PCR was performed in a 42-μl reaction mixture containing 2 μl of DNA template (or nuclease-free water as a negative control), 0.15 μg/μl of bovine serum albumin, 20 μl of 2 × GoTaq hot-start colorless master mix (Promega), and 10 μl of each primer (initial concentration, 3.2 pmol/μl). Thermal cycling was carried out in an Eppendorf Mastercyler under the following conditions: initial denaturation at 95 °C for 2 min followed by 25 cycles of 95 °C for 1 min, 55 °C for 1 min, and 72 °C for 1 min. After amplification, the DNA concentration was measured with the Qubit 2.0 fluorometer (Invitrogen) using the broad-range assay. Equimolar amounts of each PCR product were then pooled and purified using the QIAquick PCR purification kit (Qiagen). The pooled PCR purified sample was then paired-end sequenced on the Illumina Mi-Seq platform using a 150 cycle kit with a paired-end 80-bp run at the London Regional Genomics Center, London, Ontario, Canada, following standard operating procedures. (iii) Sequence processing and taxonomic assignment: Perl and Bash-based scripts were used to demultiplex the reads and assign barcoded reads to individual samples. Multiple layers of filtering were employed: (i) paired-end sequences were overlapped with Pandaseq, allowing 0 mismatches in the overlapped reads; (ii) reads were kept if the sequence included a perfect match to the V6 16S rRNA gene primers; (iii) barcodes were 8-mers with an edit distance of >4, and reads were kept if the sequence were a perfect match to the barcode; (iv) reads were clustered by 97% identity into operational taxonomic units (OTUs) using the Uclust algorithm of USEARCH version 7^57^, which has a de novo chimera filter built into it; and (v) all singleton OTUs were discarded, and those that represented ≥2% of the reads in at least one sample were kept (a filter for PCR and environmental controls and the skin swabs). Taxonomic assignments for each OTU were made by extracting the best hits from the SILVA database^58^ and then manually verified using the Ribosomal Database Project (RDP) SeqMatch tool (http://rdp.cme.msu.edu/) and using BLAST against the Greengenes database (http://greengenes.lbl.gov). Taxonomy was assigned based on hits with the highest percentage identities and coverage. If multiple hits fulfilled this criterion, classification was reassigned to a higher common taxonomy.

### Statistics and reproducibility

Data analysis for the acquired lipidomic data was performed using SIMCA (v.16; Sartorius). Orthogonal projection to latent structures-discriminant analysis (OPLS-DA) was applied to the processed Pareto-scaled, log-transformed data. Model validation was carried out using cross-validation-analysis of variance (CV-ANOVA) testing. For putative biomarker extraction, appropriate feature selection, and lipid structure annotation of candidate biomarkers, the workflow described by Vorkas et al.^17^ was followed.

Integrated analysis of the microbial and lipidomic data was performed using Pearson’s correlation coefficients and evaluated with and without FDR correction. Multivariate methods were used to identify discriminant features from the high dimensional data sets between breast cancer patients and healthy controls, namely sparse generalized canonical correlation discriminant analysis via the Data Integration Analysis for Biomarker discovery using Latent cOmponent (DIABLO) framework. This is part of the mixOmics R package. Sparse-partial least-squares-discriminant analysis (sPLS-DA) was used to integrate the relative abundance OTU data set and the lipidomics data set to classify the two groups of tissues and select the most discriminatory features from each data set. Tuning of sPLS-DA parameters was performed to determine the main OTUs and lipids that allow for the best discrimination between the healthy and diseased states with the lowest possible error rate. This resulted in a selection of 30 OTUs and 19 lipid species. The results from the sPLS-DA models were visualized using scores, loadings and circos plots that exhibit the strongest positive and negative Pearson’s correlations (|*r*| > 0.5) between the most discriminant OTUs and lipids for each subset of data and tissue types.

To compare the relative abundances of genera the ALDEx2 R package^59^ was used. The obtained values represent the expected values of 128 Monte-Carlo instances of CLR-transformed data. Effect sizes were calculated, and statistical significance was determined upon Benjamini−Hochberg correction of *P* values obtained from the Wilcoxon rank test (significance threshold *P* < 0.05).

The Kyoto Encyclopedia of Genes and Genomes (KEGG)^18–20^ was used to identify the enzymes responsible for the biosynthesis of ceramides (pathway module: M00094), diacylglycerols (entries: C00641 and C00165), phosphatidylcholines (entry: C00157), and phosphatidylethanolamines (entry: C00350). The enzymes required for the biosynthesis of lysophosphatidylcholines were identified based on a literature search. Then, the EnzymeDetector^22^ tool of the enzyme repository BRENDA^23^ (www.brenda-enzymes.org) was used to evaluate if the genes responsible for the synthesis of the enzymes of interest had been annotated in the genomes of strains of *Acinetobacter, Bacillus, Corynebacterium, Lactococcus, Prevotella, Pseudomonas*, and *Streptococcus*. Ceramidase, which is in charge of the degradation of ceramides, was also included in the analysis. A heatmap was generated using the ggplot2 package in R (Fig. 4), with categorical values denoting the presence or absence of the genes responsible for the enzymes of interest.

### Reporting summary

Further information on research design is available in the Nature Research Reporting Summary linked to this article.

## Supplementary information

Supplementary Information

Description of Additional Supplementary Files

Supplementary Data

Reporting Summary

## Data Availability

The raw data generated during the current study are available from the corresponding author on reasonable request. The processed lipidomics and microbiome data used in the figures presented in the manuscript are supplied as Supplementary Data.

## Code availability

Integrative analyses between lipidomics and microbiome data were performed using the mixOmics suite of tools (https://github.com/mixOmicsTeam/mixOmics).
