# Supplementary material for: Characterizing the breast cancer lipidome and its interaction with the tissue microbiota
Source: Commun Biol. 2021 Oct 27;4:1229. doi: 10.1038/s42003-021-02710-0 (PMC8551188; doi:10.1038/s42003-021-02710-0)
Supplement: Supplementary file 1 — Supplementary Information [file 42003_2021_2710_MOESM1_ESM.pdf]

Supplementary Table S1. Clinical data. Ductal carcinoma in situ (DCIS)

Subject	Age	Tissue type	Type of tumour	Stage of cancer	Parous	Menarche
**BTS1**	61	normal adjacent	DCIS	0	n	post
**BTS2**	87	normal adjacent	invasive lobular	1	y	post
**BTS4**	64	normal adjacent	invasive lobular	1	y	post
**BTS5**	73	normal adjacent	invasive ductal	1	y	post
**BTS6**	60	normal adjacent	invasive ductal	1	y	post
**BTS8**	68	normal adjacent	invasive ductal	1	y	post
**BTS11**	90	normal adjacent	Invasive lobular	2	y	post
**BTS12**	71	normal adjacent	DCIS	0	y	post
**BTS15**	60	normal adjacent	Invasive ductal	1	y	post
**BTS16**	54	normal adjacent	invasive	3	unknown	post
**BTS17**	58	normal adjacent	Invasive	2	unknown	post
**BTS18**	68	normal adjacent	Invasive lobular	2	unknown	post
**BTS20**	66	normal adjacent	DCIS	0	y	post
**BTS22**	67	normal adjacent	invasive ductal	3	y	post
**BTS23**	63	normal adjacent	DCIS (multifocal)	0	y	post
**BTS24**	57	normal adjacent	DCIS	0	y	post
**BTS26**	73	normal adjacent	invasive ductal	2	y	post
**BTS27**	80	normal adjacent	invasive lobular	3	y	post
**BTS28**	74	normal adjacent	Invasive ductal	1	y	post
**BTS31**	62	normal adjacent	invasive ductal	4	y	post
**BTS32**	50	normal adjacent	invasive ductal	2	n	pre
**BTS34**	70	normal adjacent	invasive ductal	1	y	post
**BTS35**	65	normal adjacent	DCIS	0	y	post
**BTS36**	54	normal adjacent	invasive ductal	1	unknown	post
**BTS37**	59	normal adjacent	invasive ductal	3	y	post
**BTS38**	66	normal adjacent	Invasive ductal	1	y	post

**BTS39**	54	normal adjacent	Infiltrating mammary carcinoma with lobular features	1	n	post
**BTS40**	69	normal adjacent	invasive mammary carcinoma	1	y	post
**BTS41**	58	normal adjacent	invasive mammary carcinoma	1	y	post
**BTS42**	47	normal adjacent	invasive mammary carcinoma	2	y	pre
**BTS43**	68	normal adjacent	invasive mammary carcinoma	1	y	post
**BTS45**	61	normal adjacent	invasive mammary carcinoma	2	y	post
**BTS46**	69	normal adjacent	invasive mammary carcinoma	1	y	post
**BTS48**	82	normal adjacent	Invasive mammary carcinoma	2	n	post
**BTS50**	57	normal adjacent	invasive lobular	3	y	post
**BTS51**	58	normal adjacent	invasive mammary carcinoma, tubular features	2	y	post
**BTS53**	51	normal adjacent	encapsulated papillary carcinoma	0	y	pre
**BTS54**	80	normal adjacent	invasive mammary carcinoma	1	y	post
**BTS55**	46	normal adjacent	invasive mammary carcinoma, tubular features	2	y	pre
**BTS56**	60	normal adjacent	invasive mammary carcinoma	2	y	post
**BTS57**	73	normal adjacent	invasive mammary carcinoma	1	y	post
**BTS58**	75	normal adjacent	metastatic lung cancer 4 yrs prior	n/a	y	post
**HS1**	47	normal	N/A (breast reductions)	N/A (healthy)	y	pre
**HS2**	42	normal	N/A (breast augmentation)	N/A (healthy)	unknown	pre
**HS3**	21	normal	N/A (breast reductions)	N/A (healthy)	n	pre
**HS4**	53	normal	N/A (breast reductions)	N/A (healthy)	unknown	unknown
**HS5**	46	normal	N/A (breast reductions)	N/A (healthy)	y	pre
**HS6**	45	normal	N/A (breast reductions)	N/A (healthy)	y	pre
**HS7**	61	normal	N/A (breast reductions)	N/A (healthy)	y	post
**HS8**	55	normal	N/A (breast reductions)	N/A (healthy)	y	pre
**HS10**	39	normal	N/A (breast reductions)	N/A (healthy)	y	pre
**HS11**	54	normal	N/A (breast reductions)	N/A (healthy)	unknown	post
**HS12**	56	normal	N/A (breast reductions)	N/A (healthy)	y	post
**HS13**	58	normal	N/A (breast reductions)	N/A (healthy)	y	post
**HS14**	59	normal	N/A (breast reductions)	N/A (healthy)	y	post
**HS15**	52	normal	N/A (breast reductions)	N/A (healthy)	y	post
**HS16**	22	normal	N/A (breast augmentation)	N/A (healthy)	n	pre
**HS17**	57	normal	N/A (breast reductions)	N/A (healthy)	y	post

**HS18**	32	normal	N/A (breast reductions)	N/A (healthy)	y	pre
**HS19**	54	normal	N/A (breast reductions)	N/A (healthy)	y	post
**HS20**	69	normal	N/A (breast reductions)	N/A (healthy)	y	post
