# Supplementary material for: Characterizing the breast cancer lipidome and its interaction with the tissue microbiota
Source: Commun Biol. 2021 Oct 27;4:1229. doi: 10.1038/s42003-021-02710-0 (PMC8551188; doi:10.1038/s42003-021-02710-0)
Supplement: Supplementary file 2 — Description of Additional Supplementary Files [file 42003_2021_2710_MOESM2_ESM.pdf]

Description of Additional Supplementary Files

File name: Supplementary Data.

Description: Processed lipidomics and microbiome data used in the figures.
