# Supplementary material for: Characterizing the breast cancer lipidome and its interaction with the tissue microbiota
Source: Commun Biol. 2021 Oct 27;4:1229. doi: 10.1038/s42003-021-02710-0 (PMC8551188; doi:10.1038/s42003-021-02710-0)
Supplement: Supplementary file 4 — Reporting Summary [file 42003_2021_2710_MOESM4_ESM.pdf]

nature research

Corresponding author(s): Jonathan R. Swann

Last updated by author(s): Jul 8, 2021

Reporting Summary

Nature Research wishes to improve the reproducibility of the work that we publish. This form provides structure for consistency and transparency in reporting. For further information on Nature Research policies, see our Editorial Policies and the Editorial Policy Checklist.

Statistics

For all statistical analyses, confirm that the following items are present in the figure legend, table legend, main text, or Methods section.

n/a	Confirmed	
☐	☒	The exact sample size (n) for each experimental group/condition, given as a discrete number and unit of measurement
☐	☒	A statement on whether measurements were taken from distinct samples or whether the same sample was measured repeatedly
☐	☒	The statistical test(s) used AND whether they are one- or two-sided<br>*Only common tests should be described solely by name; describe more complex techniques in the Methods section.*
☐	☒	A description of all covariates tested
☐	☒	A description of any assumptions or corrections, such as tests of normality and adjustment for multiple comparisons
☐	☒	A full description of the statistical parameters including central tendency (e.g. means) or other basic estimates (e.g. regression coefficient) AND variation (e.g. standard deviation) or associated estimates of uncertainty (e.g. confidence intervals)
☒	☐	For null hypothesis testing, the test statistic (e.g. F, t, r) with confidence intervals, effect sizes, degrees of freedom and P value noted<br>*Give P values as exact values whenever suitable.*
☒	☐	For Bayesian analysis, information on the choice of priors and Markov chain Monte Carlo settings
☒	☐	For hierarchical and complex designs, identification of the appropriate level for tests and full reporting of outcomes
☐	☒	Estimates of effect sizes (e.g. Cohen's d, Pearson's r), indicating how they were calculated

Our web collection on statistics for biologists contains articles on many of the points above.

Software and code

Policy information about availability of computer code

Data collection	R, Microsoft Excel
Data analysis	- Data extraction from the UPLC-MS based lipidomic profiling was conducted using the XCMS package54 (version 1.34.0) in R programming language (version 2.15.2).<br>- Data analysis for the acquired lipidomic data was performed using SIMCA (v.16; Sartorius).<br>- Integrative analyses between lipidomics and microbiome data were performed using the mixOmics (version 6.12.2) suite of tools (https://github.com/mixOmicsTeam/mixOmics) on R (version 4.0.2).<br>- For the analysis of differential bacterial abundance the ALDEx2 package (version 1.20.0) was used on R (version 4.0.2).<br>- Univariate statistical analyses, correlations and heatmaps were built using built in R functions

For manuscripts utilizing custom algorithms or software that are central to the research but not yet described in published literature, software must be made available to editors and reviewers. We strongly encourage code deposition in a community repository (e.g. GitHub). See the Nature Research guidelines for submitting code & software for further information.

Data

Policy information about availability of data

All manuscripts must include a data availability statement. This statement should provide the following information, where applicable:

- Accession codes, unique identifiers, or web links for publicly available datasets
- A list of figures that have associated raw data
- A description of any restrictions on data availability

The datasets generated and analyzed during the current study are available from the corresponding author on reasonable request

Field-specific reporting

Please select the one below that is the best fit for your research. If you are not sure, read the appropriate sections before making your selection.

☒ Life sciences ☐ Behavioural & social sciences ☐ Ecological, evolutionary & environmental sciences

For a reference copy of the document with all sections, see nature.com/documents/nr-reporting-summary-flat.pdf

Life sciences study design

All studies must disclose on these points even when the disclosure is negative.

Sample size	Sample size was calculated in R using the "pwr" package. The sample size was determined to be at least 18 subjects in each of the two groups we had (healthy and breast cancer), if we wanted to obtain at least 90% power and 5% statistical significance, with a small effect size.
Data exclusions	The population used in the current study originated a previous study (Urbaniak et al. 2014, Appl Environ Microbiology). Lipidomics profiling was only performed when adequate sample (breast tissue) was available. For the integrative part of the study were lipidomics data were interrogated against microbiome data only samples for which both datasets were available.
Replication	Replication was not performed for lipidomics analysis due to availability of starting material (breast tissue).
Randomization	This is not relevant as this is an observational study. Breast tissue samples were collected from patients who were diagnosed as having breast cancer and were thus allocated to the "breast cancer" group. Breast tissue samples from women who had no breast cancer and were undergoing surgery for cosmetic reasons were allocated to the "healthy" group. Randomization was only employed during sample preparation and lipidomics data acquisition.
Blinding	This is not relevant as this is an observational study.

Reporting for specific materials, systems and methods

We require information from authors about some types of materials, experimental systems and methods used in many studies. Here, indicate whether each material, system or method listed is relevant to your study. If you are not sure if a list item applies to your research, read the appropriate section before selecting a response.

Materials & experimental systems

n/a	Involved in the study	
☒	☐	Antibodies
☒	☐	Eukaryotic cell lines
☒	☐	Palaeontology and archaeology
☒	☐	Animals and other organisms
☐	☒	Human research participants
☒	☐	Clinical data
☒	☐	Dual use research of concern

Methods

n/a	Involved in the study	
☒	☐	ChIP-seq
☒	☐	Flow cytometry
☒	☐	MRI-based neuroimaging

Human research participants

Policy information about studies involving human research participants

Population characteristics	Participants were all women, over the age of 18. The mean age for the healthy group was 49 and for the breast cancer group, it was 65. The healthy women enrolled in the study did not have breast cancer nor a history of breast cancer and were undergoing breast surgery for cosmetic reasons. The breast cancer women enrolled in the study were women who were diagnosed with breast cancer and were undergoing surgery to either remove the tumour or more extensive mastectomies.
Recruitment	When patients were scheduled for their surgeries (either cosmetic or for breast cancer treatment), the study coordinator discussed the study with the patients, outlining all the pertinent information in the informed consent. The patients were given time to think about the study, read over the informed consent and ask the study coordinator and/or surgeon any questions. When the patient agreed to participate, the informed consent was signed. No biases were present during the recruitment process.
Ethics oversight	Ethical approval was obtained from the Western Research Ethics Board and Lawson Health Research Institute, London, Ontario, Canada.

Note that full information on the approval of the study protocol must also be provided in the manuscript.
